# Presystolic Wave is Associated with Subclinical Left Ventricular Dysfunction Assessed by Myocardial Performance Index in Type 2 Diabetes Mellitus

**DOI:** 10.5935/abc.20190134

**Published:** 2019-08

**Authors:** Selim Kul, İhsan Dursun, Semiha Ayhan, Muhammet Rasit Sayin, Özge Üçüncü, Nilgün Esen Bülbül, Ahmet Hakan Ateş, Ali Rıza Akyüz

**Affiliations:** 1 Trabzon Ahi Evren Gogus Kalp Ve Damar Cerrahisi Egitim Ve Arastirma Hastanesi - Cardiology, Trabzon - Turkey; 2 Trabzon Kanuni Egitim Ve Arastirma Hastanesi - Endocrinology, Trabzon - Turkey; 3 Trabzon Ahi Evren Gogus Kalp Ve Damar Cerrahisi Egitim Ve Arastirma Hastanesi - Internal Medicine, Trabzon - Turkey; 4 Samsun Egitim ve Arastirma Hastanesi - Cardiology, Samsun - Turkey

**Keywords:** Heart/physiopathology, Diabetes Mellitus Type 2, Ventricular Dysfunction,Left, Heart Failure, Risk Factors

## Abstract

**Background:**

Myocardial performance index (MPI), demonstrates both systolic and diastolic functions of the left ventricle. Presystolic wave (PSW) is frequently detected on Doppler examination of the left ventricular outflow tract and possible mechanism of PSW is impaired LV compliance and left ventricular stiffness.

**Objective:**

To investigate the relationship between PSW and MPI in type 2 diabetic patients.

**Method:**

A total of 129 type 2 diabetic patients were included in this study. Patients were divided into two groups according to the presence of PSW on Doppler echocardiography. There were 90 patients (38 male, mean age 57.77 ± 10.91 years) in the PSW-positive group and 39 patients (13 male; mean age: 55.31 ± 11.29 years) in the PSW-negative group. The p values of < 0.05 were considered statistically significant.

**Results:**

MPI was higher in PSW- positive group (0.63 ± 0.17vs 0.52 ± 0.13, p < 0.001). In addition, subclinical left ventricle dysfunction (LVD) was higher in the PSW- positive group (p = 0.029). Univariate analysis showed that the presence of PSW associated with abnormal MPI (p = 0.031). Pearson correlation analysis showed that PSW velocity correlated with MPI (r: 0.286, p = 0.006).

**Conclusion:**

Presence of the PSW on Doppler examination was associated with subclinical LV dysfunction in patients with DM type 2. This easy-to-perform echocardiographic parameter may be related to subclinical LVD among patients with type 2 DM.

## Introduction

Diabetic cardiomyopathy is a common, albeit frequently missed, clinical entity affecting even asymptomatic patients with type 2 diabetes mellitus (DM).^1^ These patients suffer excessive left ventricular (LV) enlargement, which starts as a normal functional consequence but later progresses to subclinical LV dysfunction (LVD).^2,3^ Patients with type 2 DM actually suffer subclinical LVD at variable rates between 25% and 60%.^4-6^ The earliest stages of diabetic cardiomyopathy are reportedly characterized by both subclinical LV systolic dysfunction (LVSD) and subclinical LV diastolic dysfunction (LVDD).^7-9^ Importantly, the latest studies have indicated a continuum in the progress of subclinical LVD despite fine glycemic control over a period of 5 years.^10^ This phenomenon may be indicative of a heightened risk of new-onset heart failure in even well-controlled type 2 DM.^11^

First defined by Tei et al.,^12^ myocardial performance index (MPI) is a surrogate marker of both ventricular systolic and diastolic functions. Its utility has been investigated in a variety of cardiac conditions including myocardial infarction, hypertension (HT), diabetes, and heart failure, and an increased MPI is reportedly an ominous prognostic sign and independent predictor for morbidity and mortality.^13,14^ A presystolic wave (PSW) is commonly found when the LV outflow tract (LVOT) is examined with the Doppler examination.^15^ PSW may theoretically be associated with poor LV compliance and increased LV stiffness.^16,17^ Given the hypothetical link between PSW and subclinical LVD in type 2 DM, we theorized that PSW may be associated with subclinical LVD in patients with type 2 DM.

## Methods

### Study population

Patients with Type 2 DM who were referred to the Cardiology and Endocrinology clinic of the Trabzon Kanuni Education and Research Hospital were enrolled by the study. A total of 129 patients were included in this study consecutively. Patients were divided into two groups according to the presence of PSW on Doppler echocardiography. There were 90 patients (38 male, mean age 57.77 ± 10.91 years) in the PSW- positive group and 39 patients (13 male; mean age: 55.31 ± 11.29 years) in the PSW-negative group. Demographic characteristics, biochemical parameters, and echocardiographic characteristics of the patients were compared between the groups. The following subjects were excluded: those with a history of hypertrophic obstructive cardiomyopathy, angina pectoris, recent myocardial infarction, coronary artery bypass surgery, peripheral arterial disease, cardiac failure, moderate to severe valvular heart disease, valvular operation, history of stroke and transient ischemic attack, atrial fibrillation, chronic renal failure, chronic liver diseases, hematological disorders, malignancy, thromboembolic disorders, congenital heart disease, congestive heart failure, and acute bacterial endocarditis. The study was approved by the local ethics committee, and all patients provided informed consent.

### Cardiovascular risk factor assessment

History of arterial HT, DM, hyperlipidemia (HL), and smoking, as well as a family history of coronary artery disease (CAD), were recorded for all patients. Type 2 DM was diagnosed on the basis of a history of treated DM and/or had a fasting blood glucose level equal to or greater than 126 mg/dl. HL was considered to exist when fasting total cholesterol level was ≥ 200 mg/dl, fasting low-density lipoprotein level ≥ 160 mg/dl, fasting triglyceride (TG) level ≥ 200 mg/dl, or using medication for HL. HT was said to be present in the case of a history of treated or untreated HT or when a mean systolic blood pressure of ≥ 140 mmHg and/or a mean diastolic blood pressure of ≥ 90 mmHg were obtained by averaging two blood pressure readings taken from each arm. The family history of CAD included a history of CAD or sudden cardiac death in first-degree male relative younger than 55 years or a first-degree female relative younger than 65 years.

### Echocardiography

**Figure 1:**
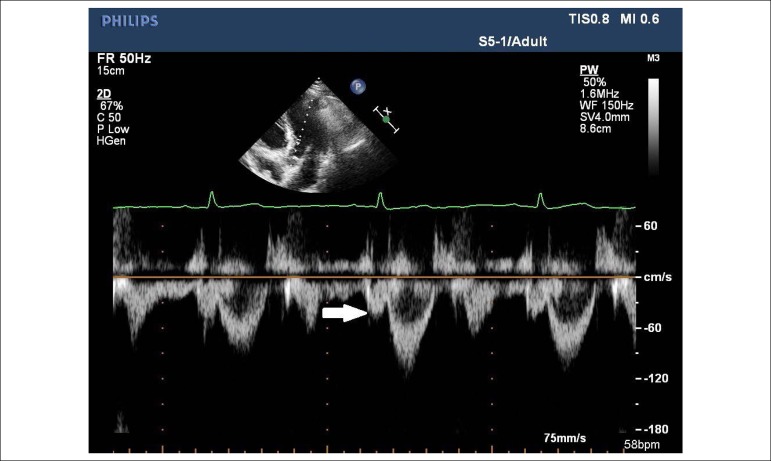
Arrow shows the PSW. PSW, presystolic wave.

**Figure 2:**
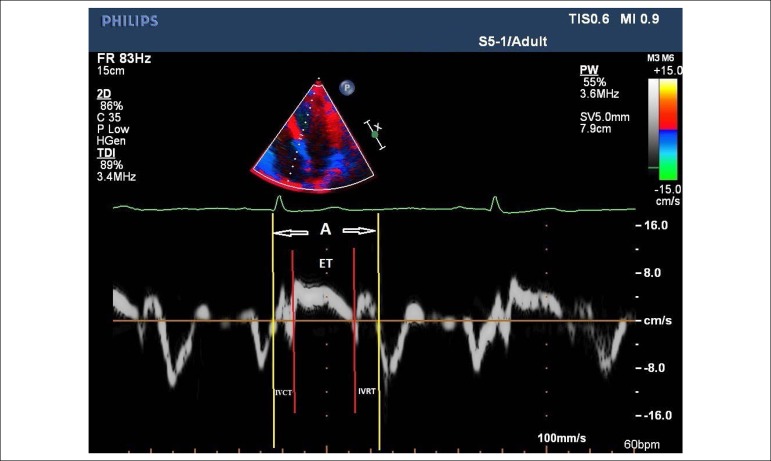
Myocardial performance index calculation. ET: ejection time; IVCT: isovolumetric contraction time; IVRT: isovolumetric relaxation time; A - time spent between closure and reopening of tricuspid valve. MPI = (IVCT+IVRT)/ET = ((A)-(ET))/(ET). MPI: myocardial performance index.

All subjects underwent a transthoracic echocardiographic examination using the Philips Epic 7 system (Philips Epic 7 Ultrasound AS) unit with a 2.5 MHz FPA probe. The conventional M-mode, B-mode, and Doppler parameters were done in compliance with the American Society of Echocardiography guidelines.^18^ All echocardiographic examinations were performed by an experienced echocardiographer who was unaware of the patients’ clinical and demographic data. Quantification of LV end-diastolic and end-systolic diameters and posterior and septal wall thicknesses were carried out. The Devereux equation was used to derive LV mass (LVM): LVM = 0.8 × [1.04 (LVEDD + IVST + PWT)^3^-(LVEDD^3^)] + 0.6, where LVEDD denotes LV end-diastolic diameter, IVST denotes intraventricular septal wall thickness, and PWT denotes posterior wall thickness. The LVM index was calculated by the formula: LVM/body surface area. Body surface area (BSA) was calculated using the ‘BSA (m^2^) = 0.007184 x Height (cm)^0.725^ x Weight (kg)^0.425^ ‘ formula. LV hypertrophy was considered positive if LVM index was above 115 g/m^2^ for men and above 95 g/m^2^ for women.^19^ LVOT’s portion just proximal to the aortic valve was interrogated with pulsed wave Doppler in the apical five-chamber window in order to check the presence of a PSW just before the LVOT flow. PSW peak velocity was quantified whenever a quantifiable PSW signal was present Figure 1. Tissue Doppler evaluation of the left ventricle was performed from the apical four-chamber view with a frame rate of greater than 80/s. All quantifications were performed on frozen images obtained from three to five cardiac cycles. Mitral annular velocities were quantified with the sample volume being placed at the junction of the mitral valve annulus and the septal myocardial wall. Time elapsed between the end of A’ wave and the beginning of the E’ wave and between the beginning of and the end of the S wave was defined as (a) and ejection time (ET), respectively, in tissue Doppler recordings done from the apical four-chamber. MPI was calculated using the ‘MPI = (IVCT + IVRT)/ET = [(a) − (ET)]/(ET)’ formula^20,21^ (Figure 2). There existed intraobserver and interobserver variability of 3% to 5% for conventional Doppler and TDI-derived variables (PSW velocity, Em, Am, and MPI). 0.5 and over MPI level was defined as subclinical LVD.

### Statistical analyses

The minimum number of subjects required in each group was determined to be 32 in order to find a significant difference between the two groups. Type I Error = 0.05, Test Power 0.80. All statistical analyses were performed using SPSS (Statistical Package for Social Sciences) for Windows 19 (SPSS Inc. Chicago, IL, USA) software package. The continuous variables were reported as mean±SD or median (interquartile range) while the categorical variables were reported as frequency and percentage. Kolmogorov Smirnov test was used to test the distribution of the quantitative variables. Independent samples t-test was used to make inter-group comparisons for normally distributed quantitative data and Mann Whitney-U test for non-normally distributed data between. Qualitative variables were compared were with the Chi-square test. Univariate analysis was performed to assess the relations between the abnormal MPI and clinical and echocardiographic variables. Pearson correlation analysis was carried out to investigate the association between PSW peak velocity and mitral A and septal A' velocities. Spearman correlation analysis was carried out to investigate the association between PSW peak velocity and Em to Am ratio and septal E’ to A’ ratio. The confidence interval was set at 95 % and statistical significance was set at p < 0.05.

## Results

Clinical and demographic characteristics of the patients are shown in Table 1. Age, sex, HT, current smoking, dyslipidemia, and family history for CAD were similar in the PSW-positive and negative groups. There was no difference between the groups in terms of left ventricular mass (LVM), left ventricular mass index (LVMI), body mass index (BMI), BSA and duration of DM. There were no patients with LVH in both groups.

**Table 1:** Demographic and biochemical characteristics of PSW positive and negative patients with type 2 DM

Variables	PSW - negative (n = 39)	PSW- positive (n = 90)	p
Age (years)	55.31 ± 11.29	57.77 ± 10.91	0.190
Sex, male, n	13	38	0.343
Hypertension, n	16	47	0.257
Current smokers, n	3	10	0,541
Family CAD, n	3	17	0.102
Dyslipidemia, n	7	13	0.632
BMI (kg/m^2^)	30.42 ± 4.97	31.29 ± 5.80	0.423
BSA (m^2^)	1.86 ± 0.18	1.87 ± 0.15	0.847
DM year	7 ( 1-10)	7 (4-12)	0.190
LVM, gr	124.17 ± 19.95	123.51 ± 32.86	0.908
LVMI, gr/m^2^	67.37 ± 10.15	66.04 ± 16.40	0.647
**Biochemical parameters**			
Glucose, mgr/dl	173.43 ± 60.14	179.38 ± 64.60	0.760
Serum creatinine, mg/dL	0.69 ± 0.18	0.78 ± 0.17	0.108
GFR,%	103.14 ± 17.71	91.10 ± 21.85	0.065
Triglyceride,mgr/dl	145.91 ± 90.44	127.67 ± 68.20	0.481
LDL-c, mgr/dl	120.72 ± 44.32	126.85 ± 30.68	0.607
HDL-c, mgr/dl	49.70 ± 11.86	47.73 ± 10.97	0.627
HbA1c, %	8.15 ± 1.74	8.26 ± 1.83	0.844
HbA1c mmol	65.57 ± 19.03	66.87± 19.97	0.842
WBC, x10^9^/L	7.77 ± 2.12	7.47 ± 1.78	0.614
PLT, x10^9^/L	240.85 ± 63.80	244.41 ± 77.61	0.881
Hb, gr/dL	13.44 ± 1.49	13.57 ± 1.59	0.789
RDW, fL	13.6 (12.9-14.9)	13.5 (13.05-14.20)	0.863
MPV, fL	9.31 ± 1.00	8.80 ± 0.94	0.104

BMI: body mass index; BSA: body surface area; CAD: coronary artery disease; DM: diabetes mellitus; LVM: left ventricle mass; LVMI: left ventricle mass index; GFR: glomerular filtration rate; LDL-c: low-density lipoprotein cholesterol; HDL-c: High density lipoprotein cholesterol; WBC: white blood cell; Hb: hemoglobin; RDW: red distribution weight; MPV: mean platelet volume; PLT: platelet.

Biochemical parameters of the study population are shown in Table 1. Serum fasting glucose, serum creatinine, high-density lipoprotein cholesterol, low-density lipoprotein cholesterol, TG, and hemoglobin A1C were not different between both groups. There was no difference between the glomerular filtration rate of both groups. There was no difference between white blood cells, hemoglobin, platelet, mean platelet volume and red distribution width between the two groups.

The echocardiographic characteristics of the PSW-positive and negative groups are shown in Table 2. Left ventricular ejection fraction, left atrial diameter, interventricular septal diameter, S velocity, mitral E deceleration time were similar in the PSW-positive and negative groups. Left ventricular end diastolic diameter (LVEDd) and posterior wall diameter was similiar in both groups.

**Table 2:** Echocardioographic variables of PSW positive and negative patients with type 2 DM

Variables	PSW negative (n = 39)	PSW positive (n = 90)	p
LVEF, %	65 (60-65)	65 (65-65)	0.858
LVEDd, cm	4.33 ± 0.36	4.26 ± 0.39	0.338
LVESd, cm	2.61 ± 0.37	2.57 ± 0.40	0.584
LAD, cm	3.22 ± 0.40	3.27 ± 0.39	0.531
IVSd, cm	0.89 ± 0.10	0.92 ± 0.16	0.265
PWd, cm	0.86 ± 0.86	0.89 ± 0.13	0.177
S velocity, cm/sn	6.29 ± 1.23	6.39 ± 1.41	0.731
E' velocity, cm/sn	9.18 ± 2.40	7.47 ± 2.35	< 0.001
A' velocity, cm/sn	8.50 ± 1.87	10.18 ± 2.21	< 0.001
Em velocity, cm/sn	94.95 ± 17.23	80.20 ± 18.81	< 0.001
Am velocity, cm/sn	82.23 ± 14,00	91.69 ± 20.50	0.010
MEdt, msn	169.35 ± 37.39	160,32 ± 34.69	0.209
MPI	0.52 ± 0.13	0.63 ± 0.17	< 0.001
Subclinic LV dysfunction	23	70	0.029
Em to Am ratio	1.14 (1.07-1.35)	0.81 (0.72 -1.13)	< 0.001
E' to A' ratio	1.18 (0.81-1.39)	0.70 (0.56-0.85)	< 0.001

LVEF: left ventricle ejection fraction; IVSd: interventricular septal diameter; PWd: posterior wall diameter; LVEDd: left ventricle end-diastolic diameter; LVESd: left ventricle end-systolic diameter; LAD: left atral diameter; MPI: myocardial performance index; MEdt: mitral E wave deceleration time; LV: left ventricle.

**Figure 3:**
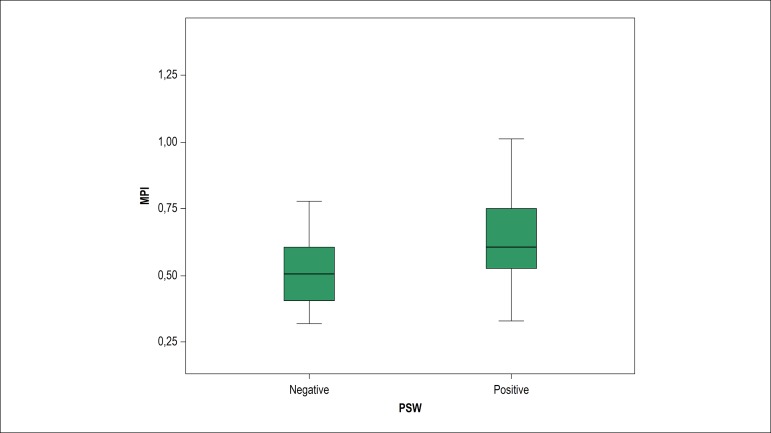
MPI level of PSW-positive and PSW-negative subjects. MPI: myocardial performance index; PSW: presystolic wave.

Doppler echocardiographic variables are shown in Table 2. Em, and septal E' wave velocities were greater in the PSW-negative group but Am and septal A' wave velocities were greater in the PSW-positive group. Em to Am ratio and septal E’ to A’ ratio were greater in the PSW-negative group. MPI was greater in the PSW-positive group (0.52 ± 0.13 vs 0.63 ± 0.17, p < 0.001) (Figure 3). Univariate analysis showed that the presence of PSW associated with abnormal MPI (p = 0.031) (Table 3). In addition, subclinical left ventricle dysfunction was more prevalent in the PSW-positive group (p = 0.029).

**Table 3:** Univariate analysis for abnormal MPI.

Variables	Odds ratios (% 95 CI)	p
Duration of DM	1.026 (0.967 - 1.089)	0.402
Age	1.014 (0.978 - 1.050)	0.445
Gender	0.818 (0.369 - 1.813)	0.621
Hypertension	2.057 (0.931 - 1.074)	0.075
Presence of PSW	2.435(1.084 - 5.466)	0.031
Hyperlipidemia	0.525 (0.195 - 1.417)	0.203
Current Smoking	1.153 (0.331 - 4.009)	0.823
Family history of CAD	4.135 (0.908 - 18.836)	0.067
BMI	1.012 (0.942 - 1.088)	0.741
Glucose	1.006 (0.995 - 1.017)	0.270
LDL-c	0.987 (0.965 - 1.009)	0.241
Trygliceride	0.999 (0.990 - 1.008)	0.812

MPI: myocardial performance index; DM: diabetes mellitus; PSW: presystolic wave; CAD: coronary artery disease; LDL-c: low-density lipoprotein cholesterol; BMI: body mass index.

**Figure 4:**
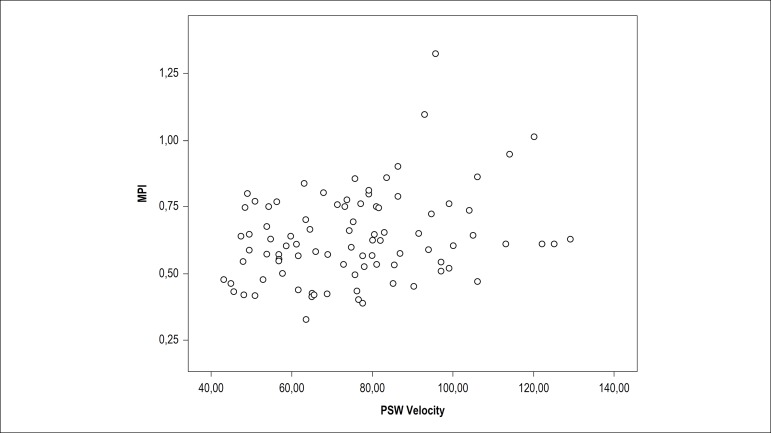
Correlation analysis between PSW velocity and MPI. PSW: presystolic wave; MPI: myocardial performance index.

The Pearson correlation analysis showed that PSW velocity was significantly correlated with mitral A wave (r: 0.402, p < 0.001) and septal A’ (r: 0.493, p < 0.001) velocities. PSW velocity was correlated with MPI (r: 0.286, p = 0.006) (Figure 4). The Spearman correlation analysis demonstrated that PSW velocity was significantly negatively correlated with Em to Am ratio (r: -0,527, p < 0.001) and septal E’ to A’ ratio (r: -0.572, p < 0.001).

## Discussion

We demonstrated an overall prevalence of PSW of 69% among type 2 DM with preserved LV ejection fraction. As compared to those without, patients with PSW had a significantly higher prevalence of subclinical LVD. Furthermore, PSW had a correlation with subclinical LVD among these patients.

A PSW is formed late in diastole commonly encountered on Doppler examination of the LVOT, which has been linked to LVDD.^16^ Mittal et al.^16^ showed a direct correlation between PSW velocity and transmitral A wave velocity; significant inverse correlation with the Em to Am ratio; and no correlation with age and LVM.^16^ Joshi et al.^22^ reported a significant correlation between PSW velocity with mitral A- wave velocity and septal A’ velocity.^21^ Among hypertensive patients, Akyuz et al.^23^ showed that PSW velocity was directly correlated with lateral A’ wave velocity and inversely with the Em to Am ratio.^23^ We detected a significant direct correlation between PSW velocity and mitral A-wave velocity, septal A’ wave velocity but there was a significant inverse relation with the mitral E to A ratio and septal E’ to A’ ratio. Akyuz et al.^23^ demonstrated a significant correlation between PSW velocity and age, LVM among hypertensive patients.^23^ Similar to Akyuz et al.,^23^ we showed a correlation between PSW velocity and age. Unlike they, however, we failed to show any correlation between PSW velocity and LVM.

As of 2015, DM affects a total of 30.3 million Americans or 9.4% of the US population. Of these individuals, 7.2 million had clinically silent DM. Moreover, 1.5 million Americans yearly are added to the diabetic population in the US.^24^ Diabetes is characterized by an increased risk of cardiovascular complications, mainly in the form of CAD, as the main source of morbidity and mortality among affected persons.^25^ Simone et al.^26^ recently reported that the risk of heart failure is heightened among type 2 diabetics and that this effect still occurs even persons do not sustain myocardial infarction or suffer HT.^26^ Hence, the term diabetic cardiomyopathy has been recommended by medical communities, referring to the dysfunctional ventricle in the absence of CAD and HT.^27^ Hyperglycemia is the source of advanced glycosylation end products (AGE). The latter are proteins with longer half-lives that have altered functional properties after being exposed to sugars and becoming glycated.^28^

When in excess, AGE formation may alter myocardial proteins structure and lead to stiff myocardium. The latter is a direct consequence of AGEs forming crosslinks between collagen molecules, which limits their degradation and leads to their accumulation in myocardial tissue, with resulting myocardial stiffness and reduced myocardial relaxation.^29^ Diabetics suffer altered myocardial function largely due to hypertrophied ventricles, metabolic abnormalities, extracellular matrix remodelling, fibrosis, vascular changes, insulin resistance, oxidative stress and apoptosis.^30,31^ Hyperglycemia may also promote myocyte apoptosis necrosis,^32^ resulting in net myocardial cell loss,^33^ reduced ventricular contractility, and systolic dysfunction. In combination, these phenomena cause reduced LV systolic and diastolic function among diabetics.

We used MPI to detect subclinical LVD. MPI is a noninvasive tool reflecting both systolic and diastolic ventricular functions, which is easy-to-perform.^34^ Its use may predict future LV impairment and development of clinical heart failure long before they become clinically apparent.^35^ It has been conclusively reported that MPI independent of blood pressure, heart rate, valvular regurgitation, ventricular geometry, preload, and afterload in patients who are lying flat.^36,37^

It is important for the clinician to determine subclinical LVD before apparent LVD occurs. For this purpose, we used MPI to identify subclinical LVD in type 2 DM. We demonstrated that MPI was significantly greater in PSW positive type 2 DM patients. This means that subclinical LVD is higher in the PSW positive group in type 2 DM. In addition, we found a correlation with PSW velocity and MPI in type 2 DM in this study. According to our study results, the presence of PSW on Doppler echocardiography and increased PSW velocity may be related to subclinical LVD in type 2 DM patients.

We did not aim to investigate the causal relationship between PSW and subclinical LVD in our study, but this relationship can be explained by several theories. The increased formation of AGEs secondary to hyperglycemia may alter structural proteins and lead to increased myocardial stiffness and impaired LV relaxation.^29^ A possible mechanism of PSW is impaired LV compliance and increased LV stiffness.^16,17^ Impaired left ventricle compliance and increased stiffness may cause the occurrence of PSW in diabetic patients. In addition, PSW is associated with LVDD.^16^ Development of LVDD may be one of the reasons for the occurrence of PSW in diabetic patients. As a result, PSW can be expected to occur in diabetic patients with subclinical LVD.

## Conclusion

Presystolic wave on echocardiography was associated with subclinical LVD in patients with DM type 2. PSW is a simple and easily detectable echocardiographic parameter seen in late diastole and may associated with subclinical left ventricle dysfunction in type 2 DM.

### Limitations of the study

Myocardial structural changes were not tested using imaging modalities. Type 2 diabetic patients alone were included in our study, limiting the use of our findings for the general population. Our findings may have been altered by antidiabetic drugs used by our patients. As our study is a cross-sectional study, its findings do fall short in making a causal relationship between MPI and PSW.
